# Birth-related characteristics predict vision-related quality of life in adults: A population-based study

**DOI:** 10.1371/journal.pone.0343620

**Published:** 2026-03-25

**Authors:** Jannet Philip, Frouke N. Boonstra, Bianca Huurneman, Nomdo M. Jansonius

**Affiliations:** 1 Royal Dutch Visio, National Foundation for the Visually Impaired and Blind, Huizen, The Netherlands; 2 Behavioural Science Institute, Radboud University, Nijmegen, The Netherlands; 3 Department of Ophthalmology, Radboud University Medical Center, Nijmegen, The Netherlands; 4 Department of Cognitive Neuroscience, Donders Institute for Brain, Cognition and Behaviour, Radboud University Medical Center, Nijmegen, The Netherlands; 5 Department of Ophthalmology, University of Groningen, University Medical Center Groningen, Groningen, The Netherlands; University of Maryland School of Medicine, UNITED STATES OF AMERICA

## Abstract

**Background:**

Individuals with an abnormal birth are at risk of having ophthalmic complications and visual impairment. The aim of this study was to understand the relationship between birth-related characteristics and vision-related quality of life (VR-QoL) in adults in the general population.

**Methods:**

We used data from Lifelines, a population-based cohort study from the North of Netherlands (n = 167,000). We included individuals with National Eye Institute Visual Function Questionnaire 25 (NEI-VFQ-25) data (n = 29,840; 18–55 years). Included birth-related characteristics were gestation length, birth weight, mode of delivery, and the presence of birth defects. We used ordinal regression with the NEI-VFQ-25 items as dependent variable and the birth-related characteristics as independent variables. Analyses were adjusted for age and gender, and corrected for multiple hypothesis testing using false discovery rate (FDR).

**Results:**

Participants with birth defects directly related to eye or brain development showed a poor VR-QoL in 9/10 subscales (FDR-corrected P value q < 0.001 to 0.05) with the most pronounced effect for difficulties performing peripheral vision-related activities. Individuals with birth defects not directly related to eye or brain development showed a poor VR-QoL in 4/10 subscales (q = 0.02 to 0.05). Early preterm birth was related to the general vision (q = 0.02) and vision specific social functioning (q = 0.05) subscales. High birth weight (>4500 g) contributed to general vision problems (q = 0.05), and vacuum or forceps mode of delivery was associated with difficulties performing activities under low luminance conditions (q = 0.02).

**Conclusion:**

At the level of the general population, birth-related characteristics are related to VR-QoL. To elucidate the underlying mechanisms, future studies could add visual function to the questionnaire outcomes.

## Introduction

Individuals with a history of abnormal birth are at an increased risk of having ophthalmic complications and visual impairment compared to adults with a history of normal birth [1–3]. Abnormal birth is defined as one or more birth-related parameters out of the normal range. Common birth measures that are assessed after birth in a new-born include gestation length, birth weight, length at birth, head and abdominal circumference, appearance, pulse, grimace, activity, and respiration (APGAR score), and physical maturity [4]. In the Netherlands, the prevalence of preterm birth (<37 weeks), recorded between 2011 and 2019, was found to be 5% [5] and the prevalence of low birth weight (<2500 grams), recorded in 2015, 6% [6]. Abnormal birth can also mean the presence of birth defects. Birth defects are structural or functional abnormalities present at birth. In the North of Netherlands, the prevalence of children born with one or more birth defects was 2.7% in 2020 [7,8]. In those newborns with a birth defect, birth defects affecting brain and eye were found in 13.8% and 7.1%, respectively [7]. Abnormal birth may cause health-related problems in adulthood and affect quality of life (QoL); it often affects visual functions as well, and thus potentially also vision-related QoL (VR-QoL) [1].

VR-QoL is a construct that is usually assessed by using questionnaires. VR-QoL refers to the degree to which impaired vision impacts daily activities or the well-being of an individual. The National Eye Institute Visual Function Questionnaire (NEI-VFQ 25) is a commonly used measure of VR-QoL. It is a 25-item questionnaire that consists of 10 vision subscales and one general health subscale [9–11].

Low gestation length (<37 weeks) and birth weight (<2500 grams) have been reported to result in smaller brain volume [12] and poorer performance on certain visual function tests such as visual acuity, contrast sensitivity, visual fields, and colour vision than in controls [12]. Previous studies reported a lower VR-QoL in adults with a history of prematurity with low birth weight compared to adults with a history of normal birth measures [2,13]. On the other tail of the distribution, children born with an abnormally high birth weight (>4500 grams) may also have long-term neuro-developmental and ocular complications [14,15].

The mode of birth delivery can have an impact on visual development and vision later in life due to perinatal damage [16,17]. Subjects born by caesarean section delivery may have a delay in cognitive development [18] and are at a higher risk for visual impairments later in life compared to those born by vaginal birth [19,20]. Vacuum and forceps-assisted births are associated with an increased risk of ocular trauma [21,22] leading to long-term visual impairments. Hence, it is possible that the mode of delivery influences VR-QoL; we could not identify studies addressing the relationship between the mode of delivery and VR-QoL.

Birth defects may cause developmental disabilities thereby leading to reduced overall quality of life [8,23]. Not only birth defects affecting the eye but also those affecting the brain may have an impact on the VR-QoL outcome. Studies conducted among individuals born with intellectual disability, hydrocephalus, and cerebral palsy showed lower scores on vision subscales of QoL questionnaires [3,24,25]. Visual problems resulting from damage to the retrogeniculate pathway are addressed as cerebral visual impairment (CVI). Of those children with birth-related developmental disabilities, 97/923 had low vision and among them 49% had CVI [26]; 6 per 1000 live births have hypoxic ischemic encephalopathy, the most common cause of CVI globally [27,28]. In the Netherlands, the prevalence of CVI in children with low vision is 27% [29].

Studying the effects of birth-related characteristics on visual functioning has direct relevance for clinical practice and health care. For example, birth-related factors can act as early ‘signals’ to identify individuals who may require targeted vision screening. Vice versa, VR-QoL questionnaires may serve as effective initial filters, helping to reduce unnecessary testing and to guide appropriate referrals. Therefore the aim of this study was to determine the relationship between various birth-related characteristics and VR-QoL in adults in the general population. For this purpose, we used data from Lifelines, a population-based cohort study conducted in the North of Netherlands.

## Methods

### Perinatal registration in the Netherlands

In the Netherlands, since 1985 information about pregnancy term, birthweight, APGAR score is stored by the national midwife registration (Landelijke Verloskunde Registratie eerste lijn;LVR1) [30]. Since 2000 the registry contains population-based information of >97% of all pregnancies in the Netherlands, data are accessible for parents. Source data are collected by 94% of midwives, 99% of gynaecologists and 68% of paediatricians, patient information is included in the registry; mothers keep documentation about birth details in a specific “groeiboekje”; currently a digital version is installed. As a result of this national perinatal care organisation, a lot of parents know the details about pregnancy term and birth weight and AGAR-score. The data in this study are based on questionnaires but participants can easily check details on their perinatal history.

### Study participants

**Fig 1:**
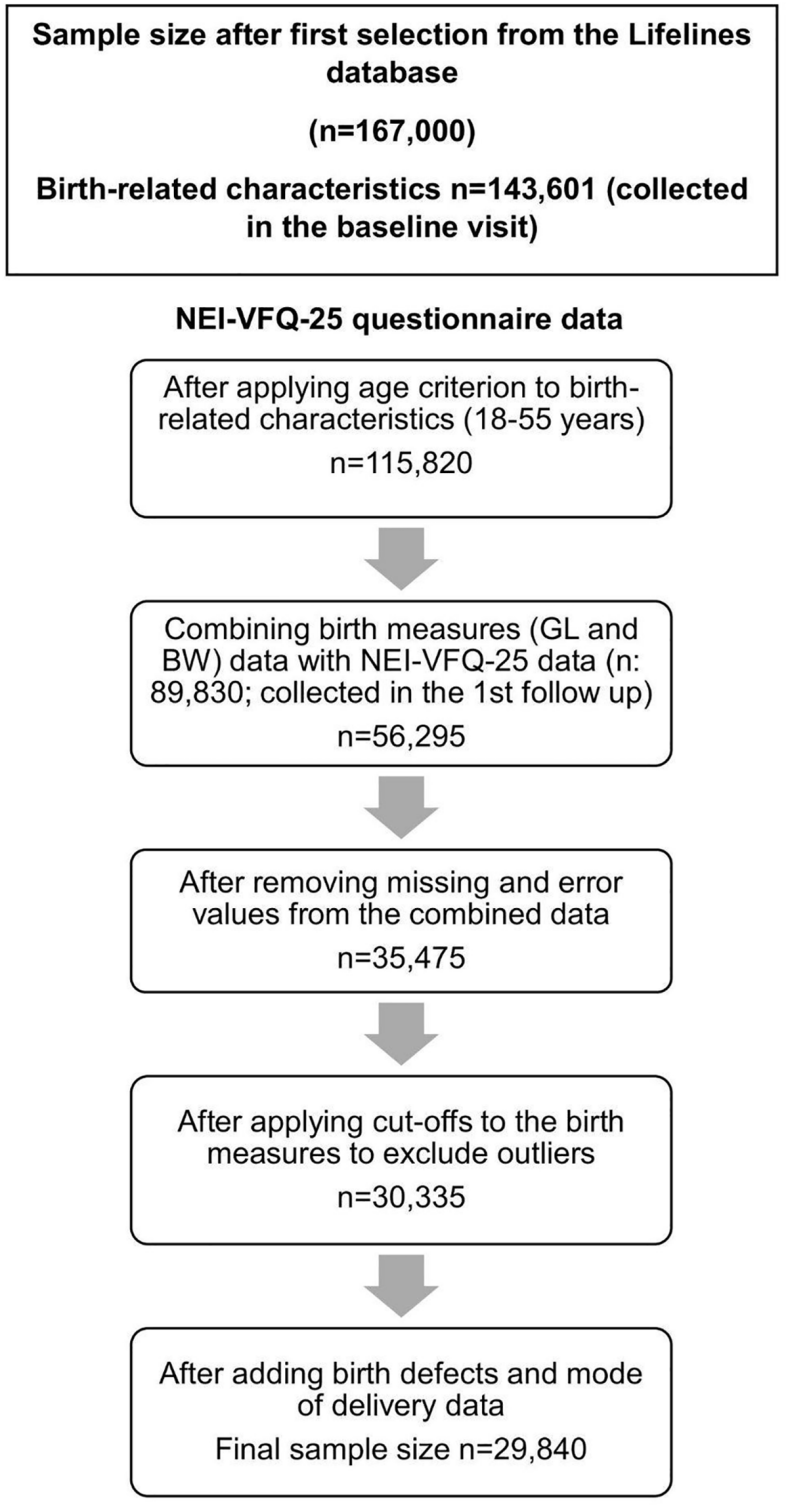
Data cleaning process of birth-related characteristics and NEI-VFQ-25 data. n: sample size, GL: gestation length, BW: birth weight.

Lifelines is a multi-disciplinary prospective population-based cohort study examining in a unique three-generation design the health and health-related behaviours of 167,729 persons living in the North of the Netherlands. It employs a broad range of investigative procedures in assessing the biomedical, socio-demographic, behavioural, physical and psychological factors which contribute to the health and disease of the general population, with a special focus on multi-morbidity and complex genetics*.* An overview of the data collection can be found in the Lifelines catalogue [31] and detailed information on the three-generation data collection is described elsewhere [32]. The baseline assessment, which includes a birth history, was conducted between 2006–2013 (n = 143,601). The NEI-VFQ-25 questionnaire was administered in participants over 17 years during the first follow-up assessment, that is, between 2014–2017 (n = 89,830 participants) [33]. In this study, we selected participants between 18–55 years of age (at the time they completed the NEI-VFQ-25 questionnaire) to study the relationship between birth-related characteristics and NEI-VFQ-25; the upper limit was set in order to minimize confounding by the presence of age-related eye diseases. Fig 1 shows the overall data cleaning process of the birth-related characteristics and the NEI-VFQ-25 data. The general Lifelines protocol has been approved by the UMCG Medical ethical committee under number 2007/152 and adheres to the tenets of the Declaration of Helsinki. A written informed consent was obtained from all participants of Lifelines.

### Vision-related quality of life

We used all items of Parts 1 and 2 of the NEI-VFQ-25 present in the Lifelines database except for the question related to general health (‘How would you describe your general health’?) [9]. In total, we used 16 items, which made up 10 subscales. The subscales were: general vision (self-rating their sight), near vision-related activities, distance vision-related activities, activities under low luminance conditions, peripheral vision-related activities, colour-vision specific activities, driving-related visual activities, vision specific social functioning, worry about sight, and ocular pain. The responses were scored according to the VFQ scoring manual [34]. In this way, the individual questions were transformed into the subscales listed above. The initial responses were between 1 (‘No difficulty at all’) and 6 (‘Stopped doing this for other reasons or not interested in doing this’). Responses 1–5 were valid, and high scores indicate poor visual function [33]. Score 6 was filtered out (except for general vision, which had 6 levels). Scores 1–5 (1–6 for general vision) were transformed into a 0–100 scale where 1 was scored as 0 and 5 as 100 (6 for general vision), with a higher score indicating a poorer VR-QoL. If a particular subscale had more than one question, an average of the transformed scores was taken and used in the analysis (to give each subscale the same range and thus facilitate a more straightforward interpretation of the odds ratios [ORs]).

### Birth-related characteristics

The birth-related characteristics were based on self-report. The variables used in this study were: gestation length (weeks), birth weight (grams), the presence of birth defects, and mode of delivery. An initial data cleaning procedure was carried out by excluding missing data and impossible values. For this, lower and upper limits were determined for gestation length and birth weight. Next, all birth-related data were transformed into categorical variables.

According to the Perinatal Dutch Registry of Netherlands (PRN) of newborn, 24 weeks was considered as the lower limit for gestation length [35]. The upper limit was taken as 43 weeks, and this was according to the Perined database in accordance with PRN [36]. Any gestation length below the 24^+0^ and above the 42^+6^ week was considered an outlier and excluded from analysis. Low gestation length (<37 weeks) was subdivided in early preterm (24–32 weeks) and late preterm (33–36 weeks). The International Fetal and Newborn Growth Consortium for the 21st Century: “ Intergrowth 21^st^ “ by the University of Oxford, an international multidisciplinary network focusing on improving perinatal and newborn growth research, was used as a reference for determining birth weight cut-off points [37,38]. Participants with a birth weight below 900 or above 6500 grams were excluded as these self-reported birth data were observed to be unreliable. This was determined graphically by assessing the scatter plots of data of birth weights of individuals below 900 and above 6500 grams against their gestation lengths, as described by Fieb et al [39]. Therefore, birth weight data between 900 and 6500 grams were used for further data analysis and categorized as low birth weight (900–2499 grams), normal (2500–4500 grams), and high birth weight (4501–6500 grams).

Mode of delivery was a categorical variable with 1–4 response options. The first 3 response options were normal vaginal birth, vaginal birth with vacuum or forceps, and caesarean birth. These 3 options were used in the analysis. Option 4 was chosen when a participant did not know their mode of delivery; these participants were excluded.

Birth defects were assessed via two questions: (1) “Did you have birth defects?” and (2) “If yes, describe the birth defect(s)”. All the responses were in Dutch. The responses were first translated into English by the first author. The first-level categorization was based on body systems. For this, the Eurocat database was used as a reference [7]. The second-level categorization was based on the type of defect. For this, we made the following categories: birth defects directly related to eye or brain development and birth defects not directly related to eye or brain development. This categorization was based on i) globally available registries on congenital birth defects [7,40,41] and ii) etiologies and co-morbidities associated with CVI [42]. Before conducting the analysis, a thorough back-translation (English to Dutch) verification of the first- and second-level categorization was undertaken by a paediatric ophthalmologist (NB). The second-level categories were used in the analysis. See Table S1 in the supplementary file S1 File for information on categorization of birth defects.

### Statistical analysis

Participants were included for analysis only if they had all the data available (complete case analysis, see Fig 1). No multicollinearity was found between the independent variables (r < 0.8).

Continuous data were described as mean and standard deviation (SD) and categorical data were given as number (percentage) for participant demographics information and birth-related characteristics data. Normality was checked using histograms where shape measures such as skewness and kurtosis were evaluated and in addition quantile-quantile plots were graphically assessed for variables before conducting analysis.

Ordinal logistic regression was performed to study the influence of the birth-related characteristics. Ordinal regression was used because the dependent variable in the models was ordinal. Dependent variables were the 10 NEI-VFQ-25 subscales; a separate model was built for each subscale. The following independent variables were entered: (1) gestation length (early preterm and not early preterm), (2) gestation length (late preterm and not late preterm), (3) birth weight (low and not low), (4) birth weight (high and not high), (5) birth defects directly related to eye or brain development and rest (including not directly related and no birth defects), (6) birth defects not directly related to eye or brain development and rest (including directly related and no birth defects), and (7) mode of delivery (vacuum or forceps, caesarean-section, and normal; each mode of delivery versus the rest). The models were adjusted for age and gender.

All statistical analysis was carried out in R Studio version 4.3.2. A p-value of 0.05 or less was considered as statistically significant. In case of multiple comparisons, the false discovery rate (FDR) method was used to calculate adjusted *P* values (*q* values). The FDR method was applied to the entire ensemble of questions (subscales for NEI-VFQ-25).

## Results

The final sample size for the first analysis addressing the association between birth-related characteristics and NEI-VFQ-25 scores was 29,840. Table 1 presents the participant characteristics.

**Table 1:** Demographics and participant birth-related characteristics.

Participant characteristics	n (%)	Mean (SD)
Age (years)	–	42.7 (8.7)
Gender (female)	19,427 (65.1)	–
Ethnicity* (European ancestry)	27,666 (99.0)	–
Gestation length (weeks)	–	39.7 (1.7)
Early preterm (24–32 weeks)	348 (1.1)	–
Late preterm (33–36 weeks)	1171 (3.9)	–
Normal (37–43 weeks)	28,321 (94.9)	–
Birth weight (grams)	–	3444 (686)
Low (<2500 grams)	1566 (5.2)	–
High (>4500 grams)	1122 (3.7)	–
Normal (2500–4500 grams)	27,152 (91.0)	–
Presence of birth defects	2085 (6.9)	–
Directly related to eye or brain development	488 (1.6)	–
Not directly related to eye or brain development	1597 (5.3)	–
No birth defects	27,755 (93.0)	–
Mode of delivery	–	–
Vaginal with vacuum or forceps	1558 (5.2)	–
Caesarean section	988 (3.3)	–
Normal vaginal	27,294 (91.4)	–

SD: standard deviation, *: for 27,952.

**Fig 2:**
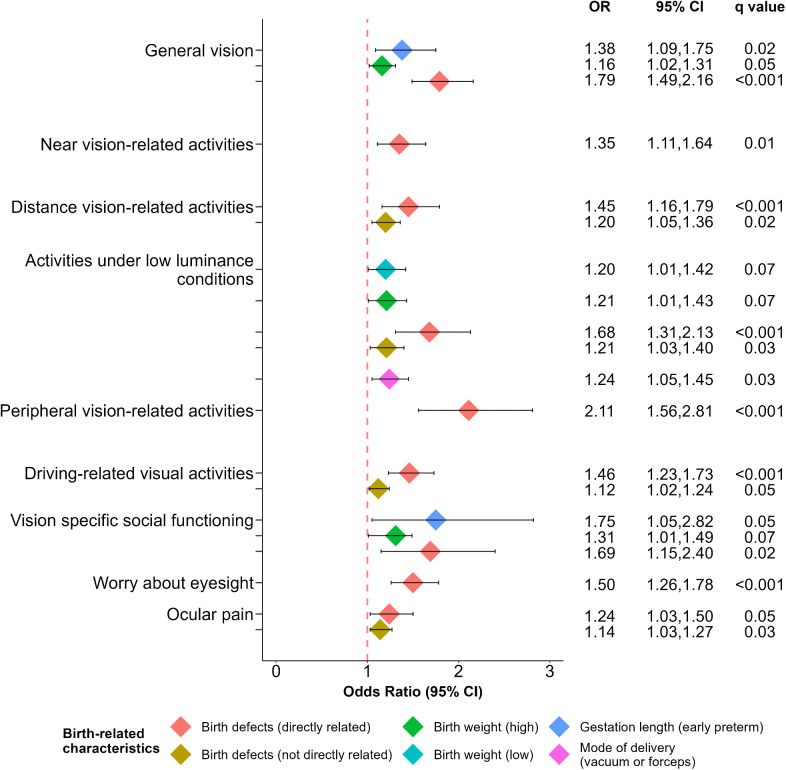
Odds ratios for birth-related characteristics that were significantly increased before correction for multiple hypothesis testing, presented per NEI-VFQ-25 subscale; the last column presents the corresponding q values (FDR adjusted P values).

Fig 2 shows the ORs of the regression models for the subscales of the NEI-VFQ-25 that were significantly associated with one or more birth-related characteristics before adjusting for multiple hypothesis testing; the last column presents the corresponding *q* values (FDR adjusted *P* values), showing that the majority but not all ORs remained significant after adjusting for multiple hypothesis testing. Color vision was not associated with any of the birth-related characteristics. The corresponding multivariable regression models for the 10 NEI-VFQ-25 subscales can be found in supplementary tables S2-S11 Tables in S1 File. Unsurprisingly, the variable that was associated with the greatest number of subscales (9/10) was birth defects directly related to eye or brain development, with the strongest association with peripheral vision-related activities (ORs ranging between 1.24 and 2.11). Here, peripheral vision was the most severely affected subscale. Interestingly, individuals with birth defects not directly related to eye or brain development also showed a poorer VR QoL, on 4/10 subscales (see Discussion section). Other variables associated with a poor VR-QoL were early preterm birth (2/10 subscales), mode of delivery (1/10), and high birth weight (1/10).

In all subscales, except for ocular pain and distance vision-related activities, age accounted for a significant amount of the variability, where an increase in age was associated with increased odds of getting a poor score on VR-QoL (ORs ranging from 1.00 to 1.11 per year increase in age). Gender had an impact on all subscales, except on near vision-related activities and peripheral vision-related activities. In the subscales colour-vision specific activities (OR 3.15) and vision specific social functioning (OR 1.56) males showed a higher risk of poor VR-QoL compared to females. In the rest of the subscales, males reported a better VR-QoL compared to females (ORs ranging from 0.31 to 0.78).

## Discussion

Birth-related characteristics are associated with VR-QoL at the level of the general population. The presence of birth defects directly related to eye or brain development was the most powerful predictor of VR-QoL. Other birth-related characteristics that predict VR-QoL were birth defects not directly related to eye or brain development, early preterm birth, high birth weight, and vacuum or forceps mode of delivery.

### Birth defects are associated with poorer VR-QoL

Birth defects directly related to eye or brain development were related to all VR-QoL subscales except for colour-vision specific activities. This is in agreement with a previous study where young adults born with birth defects due to fetal alcohol syndrome showed poor scores in all NEI-VFQ-25 subscales except for distance-vision-related activities and colour-vision specific activities [11]. Other studies have also shown a reduced VR-QoL (using other VR-QoL questionnaires) in adults with congenital neurological anomalies and developmental disabilities such as cerebral palsy, hydrocephalus, and intellectual disabilities [3,24,25]. The reduced VR-QoL in individuals with birth defects could be due to a higher number of individuals (see S1 Table in S1 File) with i) eye disorders with damages to anterior and posterior ocular structures, ii) neurological diseases, and iii) hypoxia-related conditions resulting in pre-, peri-, or postnatal damage to the brain, mainly in the higher visual centers [43,44]. Compared to the other subscales, VR-QoL for peripheral vision-related activities had a relatively high risk of being affected by birth defects (see Fig 2). This is in agreement with a study that reported visual field defects in 13.7% (351/2564) of those born with congenital eye disorders [45]. It could also be due to the presence of individuals with neurological diseases, particularly those related to hypoxia, a common cause of CVI where visual field defects are an important clinical feature [26,43].

Our results also show that people with birth defects not directly related to eye or brain development had a reduced VR-QoL in 4 out of 10 subscales. At first sight, this seems to be an unexpected finding. However, earlier studies have shown that ocular abnormalities occur frequently in individuals with congenital birth defects such as, for example, heart defects and metabolic diseases. Since our data include a large percentage of individuals with congenital heart defects and metabolic diseases (see Table S1 in S1 File, first-level categories), the associated ocular abnormalities could be one of the reasons for the observed poor VR-QoL [46,47]. Another reason could be the need to simultaneously compensate for non-visual impairments. Two of the four subscales associated with a poor VR-QoL in individuals with birth defects not related to brain or eye development were activities under low luminance conditions and driving-related visual activities. These subscales comprise visually highly demanding tasks even for healthy individuals, and the additional effort needed in the presence of non-visual impairments could have resulted in the poorer VR-QoL scores [48–50].

Thus far, VR-QoL is only studied as a part of health-related quality of life (HR-QoL) questionnaires in this population [47–50]. These studies found reduced vision scores as a part of HR-QoL, which is in agreement with our outcomes. The most commonly used method in these studies was the Health Utility Index 3, which focuses mainly on near and distance vision [51]. In our study, we found that individuals with birth defects not directly related to brain or eye development had problems with distance vision-related VR-QoL, but not with near vision-related VR-QoL.

### Early preterm birth, high birth weight, and mode of delivery and VR-QoL

Individuals born early preterm had a reduced VR-QoL in general vision and vision-specific social functioning. This is in agreement with a previous study by Kulmala et al in which reduced general vision was reported in early preterm birth compared to the outcome in full term born adults [1]. Impaired social functioning was also reported before [1,2,52]. The reduced VR-QoL could be due to the possibility of structural changes in the retina (macula and photoreceptor layer) or microstructural changes in the optic nerve and/or visual pathways and higher visual centers of the brain, in preterm [1,53–56]. Prematurity is a common causative factor of CVI in children and previous studies have reported both reduced visual functions and social functioning as a sequel to CVI due to preterm birth [29,56]. In addition, individuals born early preterm have a higher risk of co-occurring neurological conditions that may have led to difficulties in social functioning [57].

Individuals born by a vacuum or forceps mode of delivery had a reduced VR-QoL in activities under low luminance conditions. Other studies found ocular trauma and visual perceptual deficits in this group [16,20]; however, the association of vacuum or forceps mode of delivery with a reduced low luminance-related VR-QoL has not been widely studied yet. Adults born with a high birth weight had reduced VR-QoL outcomes on the general vision subscale. This agrees with a study that showed that high birth weight (>4000 grams) was positively associated with hyperopia (p < 0.001) [38]. Presence of hyperopic refractive error in individuals with high birth weight as studied earlier could be a possible explanation for a reduced score in the general vision subscale. Another possible explanation is that maternal diabetes is the major cause of high birth weight [58], and is associated with neonatal hypoglycemia, which in turn has been linked to CVI and other ocular and neurodevelopmental pathologies [59]. The observed associations between vacuum or forceps birth or high birth weight and reduced VR-QoL might also, or at least partially, be explained by unmeasured confounding factors.

Age accounted for a significant variability in 8 out of 10 subscales. These age-related effects may reflect gradual changes in visual function and the increasing visual demands of daily activities, even within this relatively young adult population. Sex differences in VR-QoL are known to be related to differences in health-reporting behaviors [60]; occupational differences and different daily visual demands may play a role as well.

### Strengths and limitations

This study showed the long-term effects of birth-related characteristics on VR-QoL. One of the major strengths of this study is the population-based study design and a large sample size. This study has some limitations as well. First, the use of self-reported birth-related characteristics – measured data are not available in Lifelines. As such, recall bias is possible. However, this will bias towards underreporting of birth-related events. As a result, true associations might be stronger than observed, and spurious associations are unlikely. To cope with this as good as possible, we followed a rigorous cleaning process by using cut-off values for each of the birth-related characteristics based on previous references. This could have caused selection bias due to over inclusion or exclusion. Secondly, there is a possibility that individuals with an abnormal birth or born with birth defects remembered their birth better than the rest, which could have introduced ‘recall bias’. Thirdly, many outcomes that we report in this study could be better understood if we could combine the outcomes with visual functional tests data that are not available in Lifelines.

### Clinical implications and future directions

Our data indicate that a significant amount of vision-related pathology can be expected in newborns with abnormal birth. Early detection of this pathology might be beneficial, either for a timely treatment or for appropriate rehabilitation. Hence, our data may alter the way we follow newborns with abnormal birth in the future. However, before we can bring these epidemiological observations to clinical guidelines or care, we need either prospective studies in newborns with and without abnormal birth (ideally) or retrospective cohort studies (more realistic).

### Conclusion

In this study, birth-related characteristics were associated with specific aspects of VR-QoL. To elucidate the underlying mechanisms, future studies could add visual function test results, and measured birth-related characteristics to the questionnaire data.

## Supporting information

S1 File S1-S11 Tables. (PDF)
