# Supplementary material for: Birth-related characteristics predict vision-related quality of life in adults: A population-based study
Source: PLoS One. 2026 Mar 25;21(3):e0343620. doi: 10.1371/journal.pone.0343620 (PMC13016338; doi:10.1371/journal.pone.0343620)
Supplement: S1 File — (PDF) [file pone.0343620.s001.pdf]

Supplementary Tables S1-S11

Table S1. Categorization of birth defects

	First level categories n (%)*	Second level categories n (%)†
CNS and brain-related anomalies	28 (1.3)	28 (100)
Congenital eye anomalies	171 (8.2)	161 (94.1)
Congenital craniofacial anomalies	49 (2.3)	39 (79.5)
Multiple organ diseases	72 (3.4)	25 (34.7)
Congenital haemolytic diseases	55 (2.6)	43 (78.1)
Congenital metabolic diseases	57 (2.7)	47 (82.4)
Neurodevelopmental anomalies and developmental disabilities	52 (2.5)	52 (100)
Hypoxia-related history	53 (2.5)	53 (100)
Syndromes and genetic anomalies	44 (2.1)	33 (75.0)
Congenital ear, mouth and spine anomalies	115 (5.5)	-
Congenital anomalies of the heart	317 (15.2)	-
Congenital hip and limb anomalies	556 (27.0)	-
Congenital respiratory system anomalies	31 (1.4)	-
Congenital anomalies of digestive system	132 (6.3)	-
Congenital anomalies of renal system	79 (4.0)	-
Congenital anomalies of the reproductive and genital system	33 (1.5)	-
Congenital nevus	62 (3.0)	-
Congenital immune system anomalies	45 (2.1)	-
Congenital tumours	20 (1.0)	-
Other congenital anomalies	114 (5.4)	7 (6.1)

Total (n)	2085	488

** : Percentage is based on total birth defects, †: percentage is based on the first level categories.*

Table S2. Multivariable regression model of general vision-related VR-QoL

Birth-related characteristic	q value	OR	95% CI
Gestation length (early preterm)	0.02	1.38	1.09-1.75
Gestation length (late preterm)	0.5	1.06	0.91-1.18
Birth weight (low)	0.2	1.11	0.98-1.25
Birth weight (high)	0.05	1.16	1.02-1.31
Birth defect (directly related)	<0.001	1.79	1.49-2.16
Birth defect (not directly related)	0.7	1.03	0.93-1.14
Mode of delivery (vacuum or forceps)	0.2	0.92	0.83-1.02
Mode of delivery (c-section)	0.4	0.94	0.82-1.07
Age	<0.001	1.06	1.05-1.06
Gender	<0.001	0.71	0.67-0.75

Table S3. Multivariable regression model of near vision-related activities VR-QoL

Birth-related characteristic	q value	OR	95% CI
Gestation length (early preterm)	0.5	1.12	0.87-1.44
Gestation length (late preterm)	0.6	1.05	0.91-1.18
Birth weight (low)	0.9	1.0	0.87-1.17
Birth weight (high)	0.4	1.06	0.94-1.21
Birth defect (directly related)	0.01	1.35	1.11-1.64
Birth defect (not directly related)	0.3	1.07	0.96-1.20
Mode of delivery (vacuum or forceps)	0.3	0.92	0.81-1.04
Mode of delivery (C-section)	0.9	1.00	0.85-1.17
Age	<0.001	1.11	1.11-1.12
Gender	0.1	1.05	0.99-1.11

Table S4. Multivariable regression model of distance vision-related activities VR-QoL

Birth-related characteristic	q value	OR	95% CI
Gestation length (early preterm)	0.3	1.19	0.89-1.57
Gestation length (late preterm)	0.5	1.07	0.91-1.26
Birth weight (low)	0.1	1.15	0.99-1.32
Birth weight (high)	0.1	1.17	1.00-1.36
Birth defect (directly related)	<0.001	1.45	1.16-1.79
Birth defect (not directly related)	0.02	1.20	1.05-1.36
Mode of delivery (vacuum or forceps)	0.9	1.00	0.87-1.15
Mode of delivery (c-section)	0.7	1.04	0.88-1.23
Age	0.07	1.00	1.00-1.00
Gender	<0.001	0.78	0.73-0.84

Table S5. Multivariable regression model of activities under low luminance conditions

Birth-related characteristic	q value	OR	95% CI
Gestation length (early preterm)	0.7	1.11	0.78-1.54
Gestation length (late preterm)	0.7	1.03	0.85-1.25
Birth weight (low)	0.07	1.20	1.01-1.42
Birth weight (high)	0.07	1.21	1.01-1.43
Birth defect (directly related)	<0.001	1.68	1.31-2.13
Birth defect (not directly related)	0.03	1.21	1.03-1.40
Mode of delivery (vacuum or forceps)	0.03	1.24	1.05-1.45
Mode of delivery (c-section)	0.1	1.21	0.99-1.48
Age	<0.001	1.05	1.04-1.06
Gender	<0.001	0.41	0.39-0.46

Table S6. Multivariable regression model of peripheral vision-related activities

Birth-related characteristic	q value	OR	95% CI
Gestation length (early preterm)	0.7	1.10	0.69-1.70
Gestation length (late preterm)	0.2	1.21	0.94-1.54
Birth weight (low)	0.2	1.27	1.01-1.57
Birth weight (high)	0.3	1.18	0.93-1.49
Birth defect (directly related)	<0.001	2.11	1.56-2.81
Birth defect (not directly related)	0.2	1.18	0.95-1.45
Mode of delivery (vacuum or forceps)	0.1	1.20	0.96-1.49
Mode of delivery (c-section)	0.9	1.03	0.76-1.36
Age	<0.001	1.05	1.04-1.05
Gender	0.4	0.94	0.85-1.05

Table S7. Multivariable regression model of colour vision specific activities

Birth-related characteristic	q value	OR	95% CI
Gestation length (early preterm)	0.5	1.19	0.59-2.22
Gestation length (late preterm)	0.6	1.19	0.80-1.70
Birth weight (low)	0.9	1.04	0.70-1.48
Birth weight (high)	0.4	1.20	0.85-1.63
Birth defect (directly related)	0.1	1.52	0.92-2.37
Birth defect (not directly related)	0.7	1.07	0.77-1.46
Mode of delivery (vacuum or forceps)	0.8	1.04	0.74-1.43
Mode of delivery (c-section)	0.9	1.00	0.62-1.52
Age	<0.001	1.05	1.04-1.06
Gender	<0.001	3.15	2.72-3.66

Table S8. Multivariable regression model of driving-related visual activities

Birth-related characteristic	q value	OR	95% CI
Gestation length (early preterm)	0.3	1.17	0.93-1.46
Gestation length (late preterm)	0.7	1.03	0.91-1.16
Birth weight (low)	0.4	1.02	0.95-1.18
Birth weight (high)	0.7	1.03	0.91-1.16
Birth defect (directly related)	<0.001	1.46	1.23-1.73
Birth defect (not directly related)	0.05	1.12	1.02-1.24
Mode of delivery (vacuum or forceps)	0.3	1.07	0.97-1.18
Mode of delivery (c-section)	0.2	0.90	0.79-1.03
Age	<0.001	1.01	1.00-1.01
Gender	<0.001	0.32	0.30-0.33

Table S9. Multivariable regression model of vision specific social functioning

Birth-related characteristic	q value	OR	95% CI
Gestation length (early preterm)	0.05	1.75	1.05-2.82
Gestation length (late preterm)	0.9	0.97	0.65-1.25
Birth weight (low)	0.5	0.87	0.63-1.18
Birth weight (high)	0.07	1.31	1.01-1.68
Birth defect (directly related)	0.02	1.69	1.15-2.37
Birth defect (not directly related)	0.3	1.18	0.91-1.49
Mode of delivery (vacuum or forceps)	0.3	1.16	0.90-1.49
Mode of delivery (c-section)	0.3	0.77	0.50-1.12
Age	<0.001	1.05	1.04-1.06
Gender	<0.001	1.56	1.39-1.75

Table S10. Multivariable regression model of worry about sight

Birth-related characteristic	q value	OR	95% CI
Gestation length (early preterm)	0.4	1.11	0.90-1.38
Gestation length (late preterm)	0.1	1.12	1.0-1.25
Birth weight (low)	0.5	0.97	0.87-1.08
Birth weight (high)	0.9	0.99	0.89-1.11
Birth defect (directly related)	<0.001	1.50	1.26-1.78
Birth defect (not directly related)	0.4	1.06	0.96-1.16
Mode of delivery (vacuum or forceps)	0.1	0.91	0.82-1.00
Mode of delivery (c-section)	0.8	1.01	0.90-1.14
Age	<0.001	1.03	1.02-1.03
Gender	<0.01	0.92	0.88-0.97

Table S11. Multivariable regression model of ocular pain

Birth-related characteristic	q value	OR	95% CI
Gestation length (early preterm)	0.1	0.79	0.60-1.01
Gestation length (late preterm)	0.9	0.99	0.87-1.14
Birth weight (low)	0.5	1.06	0.94-1.19
Birth weight (high)	0.1	1.13	0.99-1.28
Birth defect (directly related)	0.05	1.24	1.03-1.50
Birth defect (not directly related)	0.03	1.14	1.03-1.27
Mode of delivery (vacuum or forceps)	0.3	0.93	0.83-1.04
Mode of delivery (c-section)	0.7	1.02	0.89-1.17
Age	0.1	1.01	1.00-1.01
Gender	<0.001	0.67	0.63-0.71
